# Impact of Ambient Temperature and Relative Humidity on the Incidence of Hand-Foot-Mouth Disease in Wuhan, China

**DOI:** 10.3390/ijerph17020428

**Published:** 2020-01-08

**Authors:** Jiayuan Hao, Zhiyi Yang, Wenwen Yang, Shuqiong Huang, Liqiao Tian, Zhongmin Zhu, Yuanan Lu, Hao Xiang, Suyang Liu

**Affiliations:** 1 Department of Global Health, School of Health Sciences, Wuhan University, 115# Donghu Road, Wuhan 430071, China; haohaojy777@gmail.com (J.H.); 13967039772@163.com (Z.Y.); 2 Global Health Institute, Wuhan University, 115# Donghu Road, Wuhan 430071, China; 3 Hubei Provincial Center for Disease control and Prevention, Wuhan 430079, China; wenwenyanglinyi@163.com (W.Y.); hsq7513@163.com (S.H.); 4 State Key Laboratory of Information Engineering in Surveying, Mapping and Remote Sensing, Wuhan University, Wuhan 430079, China; tianliqiao@whu.edu.cn (L.T.); zhongmin.zhu@whu.edu.cn (Z.Z.); 5 College of Information Science and Engineering, Wuchang Shouyi University, Wuhan 430064, China; 6 Environmental Health Laboratory, Department of Public Health Sciences, University of Hawaii at Manoa, 1960 East-West Rd, Biomed Bldg, D105, Honolulu, HI 96822, USA; yuanan@hawaii.edu

**Keywords:** temperature, apparent temperature, relative humidity, HFMD, PM_2.5_, DLNM

## Abstract

*Background*: Few studies have previously explored the relationship between hand, foot, and mouth disease (HFMD) and meteorological factors with the effect modification of air pollution, and these studies had inconsistent findings. We therefore applied a time-series analysis assessing the effects of temperature and humidity on the incidence of HFMD in Wuhan, China to deepen our understanding of the relationship between meteorological factors and the risk of HFMD. *Methods:* Daily HFMD cases were retrieved from Hubei Provincial Center for Disease Control and Prevention from 1 February 2013 to 31 January 2017. Daily meteorological data including 24 h average temperature, relative humidity, wind velocity, and atmospheric pressure were obtained from Hubei Meteorological Bureau. Data on Air pollution was collected from 10 national air-monitoring stations in Wuhan city. We adopted a distributed lag non-linear model (DLNM) combined with Poisson regression and time-series analysis to estimate the effects of temperature and relative humidity on the incidence HFMD. *Results:* We found that the association between temperature and HFMD incidence was non-linear, exhibiting an approximate “M” shape with two peaks occurring at 2.3 °C (RR = 1.760, 95% CI: 1.218–2.542) and 27.9 °C (RR = 1.945, 95% CI: 1.570–2.408), respectively. We observed an inverted “V” shape between relative humidity and HFMD. The risk of HFMD reached a maximum value at a relative humidity of 89.2% (RR = 1.553, 95% CI: 1.322–1.824). The largest delayed cumulative effects occurred at lag 6 for temperature and lag 13 for relative humidity. *Conclusions:* The non-linear relationship between meteorological factors and the incidence of HFMD on different lag days could be used in the early targeted warning system of infectious diseases, reducing the possible outbreaks and burdens of HFMD among sensitive populations.

## 1. Introduction

Hand, foot, and mouth disease (HFMD) is a highly contagious disease transmitted by enterovirus, mainly including coxsackie virusA16 (CV-A16) and enterovirus 71 (EV-17). HFMD usually occurs among children under five years old, although sometimes adolescents and adults get infected as well [1]. The average incubation period for HFMD is from three to seven days. The main symptoms of HFMD are painful mouth ulcers, vesicles on the hands and feet, and fever [2]. Despite the fact most cases of HFMD are self-healing, some severe neurological and systemic complications may follow the infection, causing a heavy burden on public health [3,4].

In the past decades, the Asia–Pacific region has witnessed a number of HFMD epidemics. For example, Taiwan experienced an outbreak involving millions of people in 1998 [5]. In mainland China, HFMD was responsible for more than 1100 infections in Shandong in 2007 [6]. The 2008 Anhui epidemic led to 480,000 cases and 128 deaths [7]. In 2012, the annual report of the National Health and Family Planning Commission warned that both the incidence and mortality of HFMD are highest among category C infectious diseases in China [8].

Weather is considered to be a major factor affecting outbreaks of infectious diseases [9]. Studies have explored the associations between climate factors and various infectious diseases, such as tuberculosis, influenza, mumps, etc. [10,11,12]. There have been some studies on the association between meteorological factors and HFMD [13,14,15]. However, possibly due to the heterogeneity of climate factors and geographic conditions among these studies, the results were not consistent. Linear and non-linear relationships were both observed when quantifying the effects of temperature on HFMD [16,17,18]. Some studies claimed that high relative humidity contributed to an increased risk of HFMD, while others found no association [15,19,20,21,22,23]. There are relatively few studies indicating an association between wind velocity and the incidence of HFMD and the results are not consistent [20,23,24,25]. In addition, exposure to air pollution has also been found to play a role in HFMD infection [26], although such studies are rare.

Our study aims to explore the association between meteorological factors and the incidence of HFMD by controlling the effects of air pollution from 1 February 2013 to 31 January 2016 in Wuhan, China. We reported both single-day lag effects and cumulative effects. We estimated the extreme effects of temperature and humidity including the 1st, 5th, 10th, 90th, 95th, and 99th percentiles of meteorological factors. We also compared the effects of ambient temperature and apparent temperature in our sensitive analysis.

## 2. Methods

### 2.1. Study Area

Wuhan (29°58′–31°22′ N, 113°41′–115°05′ E) is the capital city of Hubei Province in central China, covering a land size of 8569.15 km^2^ and a total population of 11,360,000 in 2016 [27]. Wuhan has a typical subtropical monsoon climate, with a daily mean temperature of 17.3 °C and relative humidity of 80% during 2016. From 2013 to 2016, more than 65,000 HFMD cases were documented in Wuhan.

### 2.2. Data Collection

HFMD was categorized as a Category C infectious disease in China in 2008 and the disease surveillance is updated every day. Daily HFMD cases were retrieved from the disease surveillance system from the Disease Reporting System in Hubei Provincial Center for Disease Control and Prevention from 1 February 2013 to 31 January 2017. HFMD was diagnosed based on the clinical criteria in the “HFMD Control and Prevention Guide” published by the Chinese Ministry of Health [28].

Daily meteorological data, including 24 h average ambient temperature, relative humidity, wind velocity, vapor pressure, and atmospheric pressure, were obtained from Hubei Meteorological Bureau.

Data on air pollution were collected from 10 national air quality monitoring stations in Wuhan. Previous studies indicated that exposure to particulate matter less than or equal to 10 micrometers in aerodynamic diameter (PM_10_), particulate matter less than or equal to 2.5 micrometers in aerodynamic diameter (PM_2.5_), and sulfur dioxide (SO_2_), has the potential to increase the risk of HFMD [23,29,30]. Therefore, we included these three air pollutants in our analysis, namely 24 h concentrations of PM_10_, PM_2.5_, and SO_2_. The daily concentration of each air pollutant in Wuhan was averaged based on measurements collected from 10 monitoring stations across the city.

Apparent temperature was calculated as follows [31]:

(1) AT=−2.7 + 1.047·Ta + 2.0·Pv − 0.65·WV 

where *AT* is apparent temperature, *Ta* is ambient temperature (°C), *Pv* is vapor pressure (hPa), and *WV* is wind velocity (m/s) 10 m above the ground.

### 2.3. Statistical Analysis

Spearman correlation was used to assess the relationship between pollutant concentrations and meteorological factors. In our univariate analysis, we found that all air pollutants and meteorological factors were significantly associated with the incidence of HFMD, except for wind velocity. Therefore, this article mainly focuses on reporting the effects of temperature and relative humidity.

As the distributed lag non-linear model (DLNM) based on the generalized linear model (GLM) or generalized additive model [32] is considered to represent the time-course of the exposure–response and cumulative lag effects in a non-linear process more flexibly [33], we adopted a DLNM with Poisson regression to estimate the effects of meteorological factors on the risk of HFMD. In order to assess the exposure–lag–response relationship, we used a cross-basis function for temperature, relative humidity, and wind velocity. The model is as follows

(2) $$Log\left[E\left(y_{t}\right)\right]=\alpha +\sum cb\left(M,df,lag,df\right)+\sum ns\left(Xi\right)+ns\left(time,8\right)+\gamma DOW$$

where *t* is the day of observation; $E\left(y_{t}\right)$ is the observed number of HFMD cases on *t*; *α* is the intercept; *cb* is a cross-basis function used for estimating the non-linear relationship between meteorological factors and HFMD incidence; *M* is the examined meteorological variable that was closely linked with incidence of HFMD; *ns(X_i_)* represents a natural cubic spline function for atmospheric pressure and air pollutants; *time* is the indicator variable used to control long-term trends and seasonality; *DOW* is an indicator variable representing day of the week. The degree of freedom (df) for each variable was determined by the Akaike Information Criterion for quasi-Poisson (Q-AIC). df for *M* and their corresponding lags are as follows: 5.4 for temperature, 4.4 for relative humidity, and 3.3 for wind velocity. Atmospheric pressure, PM_2.5_, and SO_2_ were controlled for by the natural cubic spline with 5, 4, and 4 df, respectively. The knots were equally spaced for exposure–response and the log scale of lags by default. The df per calendar year for time variable was set to 8. To avoid the potential collinearity due to the presence of PM_2.5_ and PM_10_ (r = 0.869), we included only PM_2.5_ and SO_2_ in the analysis, and used PM_10_ instead in the sensitivity analysis to verify the robustness of the model.

Because the incubation period of HFMD is 2–10 days, the maximum lag day in our analysis was set to 14 days to estimate the overall cumulative effects. We also divided lag days of the study variables into four categories (lag3, lag7, lag10, and lag14) to effectively depict various single-day effects. Spline knots were defined at equal spaces and the knots of lags were also equally placed on the log scale. We selected the medium values of ambient temperature (18.4 °C) and relative humidity (78.6%) as the reference values in the DLNM model. Relative risks (RR) with 95% confidence intervals (CIs) for different percentiles (1st, 5th, 10th, 90th, 95th, and 99th) of ambient temperature and relative humidity were used to represent the risk of HFMD for extreme meteorological factors.

We applied several sensitivity analyses to test the robustness of our model. First, we altered the df for long-term trend (6 to 7 df) to find the best model fit. Second, we replaced PM_2.5_ with PM_10_ to examine the robustness of our results. Lastly, we used the apparent temperature rather than the ambient temperature to assess the effects.

Data cleaning and analysis were accomplished using R packages “dlnm” and “spline” (v.3.4.2; R Foundation for Statistical Computing, Vienna, Austria).

## 3. Results

From 1 February 2013 to 31 January 2017, a total of 65,313 HFMD cases was documented in Wuhan, China. Characteristics of meteorological factors, air pollutants, and HFMD cases are shown in Table 1. The mean value of each meteorological variable was 17.29 °C for ambient temperature, 14.62 °C for apparent temperature, 77.90% for relative humidity, 1.73 m/s for wind velocity, an2d 1012.93 hPa for atmosphere pressure. The lowest apparent temperature was much lower than the lowest ambient temperature, while the highest ambient temperature was slightly higher than the highest apparent temperature. The average concentration of each air pollutant was 109.98, 72.28, and 25.31 μg/m^3^ for PM_10_, PM_2.5_, and SO_2_, respectively. The daily average number of HFMD cases was 44.8, ranging from 0 to 239. The spearman correlation between meteorological factors and air pollution is presented in Table 2. Air pollutants were negatively correlated with ambient temperature, apparent temperature, relative humidity, and wind velocity. Except for the correlation between wind velocity and apparent temperature, all other meteorological factors and air pollutants were significantly correlated with each other.

**Figure 1:**
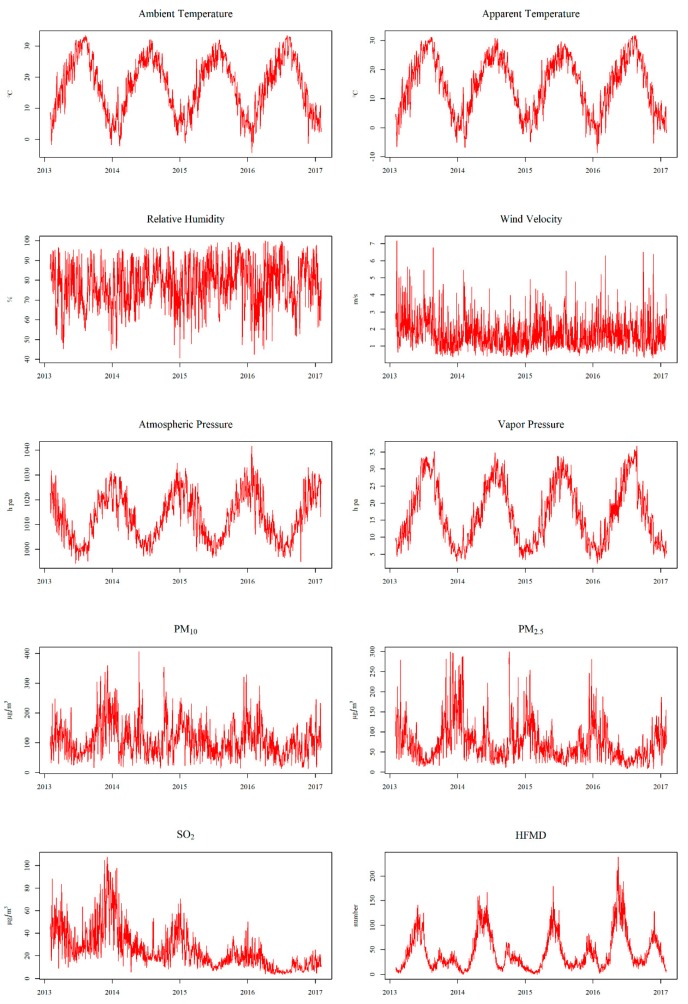
Seasonal fluctuations in meteorological factors, air pollutants, and HFMD cases in Wuhan, China from 1 February 2013 to 31 January 2017.

Figure 1 illustrates the seasonal fluctuation in meteorological factors, air pollutants, and HFMD cases. Ambient temperature, apparent temperature, atmosphere pressure, and PM_2.5_ presented a periodic pattern. The concentration of SO_2_ showed a decreasing but observable seasonal trend between 2013 and 2017. We also observed the seasonality of HFMD cases with two peaks in a year: one was during late spring to early summer (April to June) and the other was during late fall to winter (November to January), although the second one is less pronounced than the first one.

**Figure 2:**
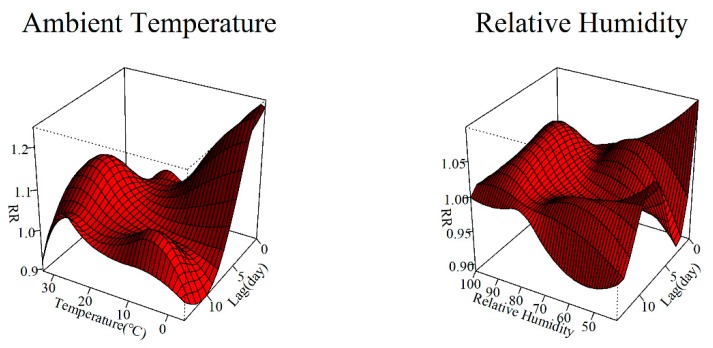
Three dimensional (3D) lag–response curves specific to ambient temperature and relative humidity.

**Figure 3:**
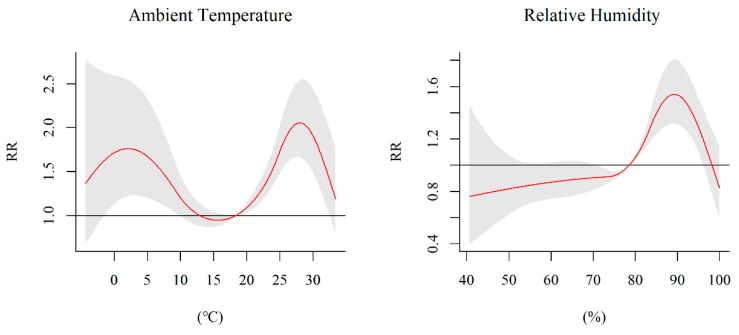
Cumulative effects of ambient temperature and relative humidity on HFMD incidence within 14 days.

We used three-dimensional (3D) plots to visualize the exposure–lag–response relationship of HFMD and climate factors (Figure 2). Figure 3 demonstrates the cumulative effects of ambient temperature and relative humidity over 14 days, taking their medium values as references. The associations between ambient temperature, relative humidity, and the risk of HFMD were non-linear. The exposure–response curve for the ambient temperature presents an ‘M’ shape with two peaks. The first peak was at 2.3 °C with an RR of 1.760 (95% CI: 1.218–2.542), and the second one was at 27.9 °C with an RR of 1.945 (95% CI: 1.570–2.408). For relative humidity, we observed an inverted ‘V’ shape that reached the maximum value at a humidity of 89.2% (RR = 1.553, 95% CI: 1.322–1.824).

**Figure 4:**
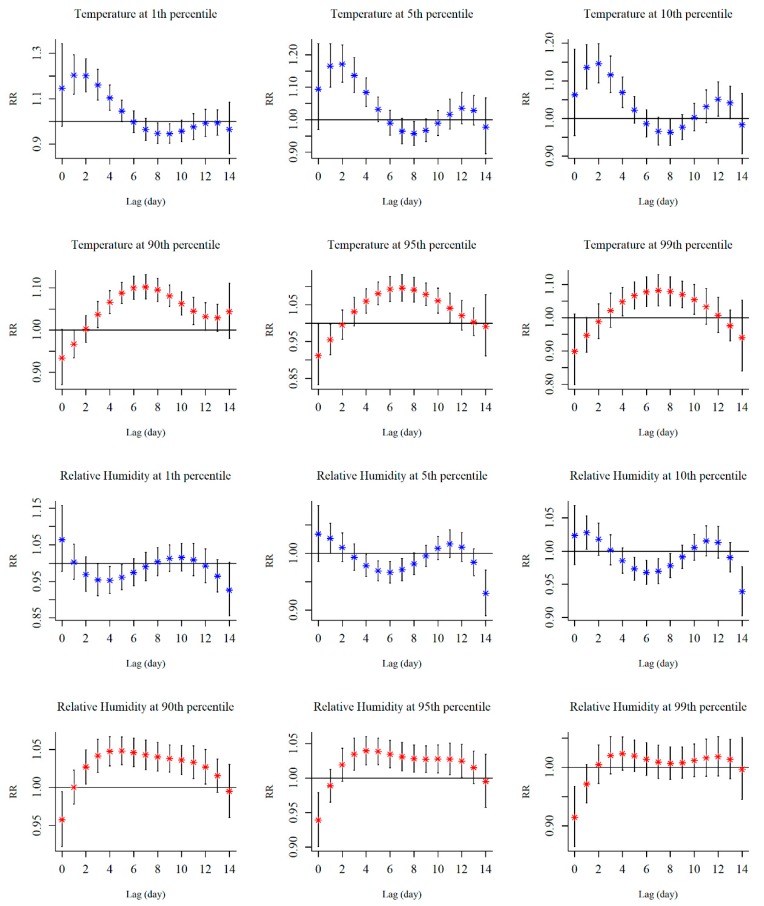
The distributed lagged effects of extreme ambient temperature and relative humidity on the incidence of HFMD at various lag days. The medium value of each variable was selected as the reference.

The associations between extreme meteorological factors and the risk of HFMD at specific lag days are shown in Figure 4. The effects of extreme low temperature peaked at lag2, then decreased and reached a second peak at lag12. The effects of extreme high temperature presented an inverted ‘V’ shape, and the peak appeared at lag7. We observed that the hot effects occurred relatively slower than the cold effects. For low temperatures, the effect estimates at the 1st percentile was greater than estimates at the 5th and 10th percentiles, while for high temperatures, the risk of HFMD at higher temperatures was lower than at lower temperatures. Low relative humidity was a significant protective factor for the risk of HFMD between lag3 to lag5 at the 90th percentile, lag4 to lag7 at the 95th percentile, and lag5 to lag8 at the 99th percentile. Relative humidity at the 90th percentile was significantly associated with an increased risk of HFMD from lag2 to lag 12. Similar with extreme high temperatures, the effects at the 90th percentile were more pronounced than those at the 99th percentile for relative humidity. The lagged period was divided at lag3, lag7, lag10, and lag14 and their effects were shown in Figure S1.

Table 3 presents the cumulative effects of extreme ambient temperature and relative humidity at lag0–14. The risk of HFMD reached the highest level at the 5th percentile for low temperature (RR = 1.756, 95% CI: 1.224–2.518), and the 90th percentile for high temperature (RR = 1.919, 95% CI: 1.530–2.407). Low relative humidity showed a protective effect for the incidence of HFMD, although it was not statistically significant and the strongest effects occurred at the 1st percentile of relative humidity (RR = 0.805, 95% CI: 0.597–1.086). Compared with the effects at the 95th percentile and the 99th percentile, the risk for the 90th percentile of the relative humidity had greater adverse effects on HFMD incidence (RR = 1.474, 95% CI: 1.261–1.722).

Table 4 shows the largest cumulative effects of meteorological factors on HFMD from lag0–1 to lag0–14. For ambient temperature, the extreme cold effects occurred at −4.3 °C from lag0–1 to lag0–10. The extreme hot weather had the greatest adverse effects, from lag0–12 to lag0–14. Different from the ambient temperature, the largest effects for the extreme low relative humidity between lag0–1 to lag0–3 were not statistically significant. High relative humidity was significantly associated with the increased risk of HFMD from lag0–4 to lag0–14.

**Figure 5:**
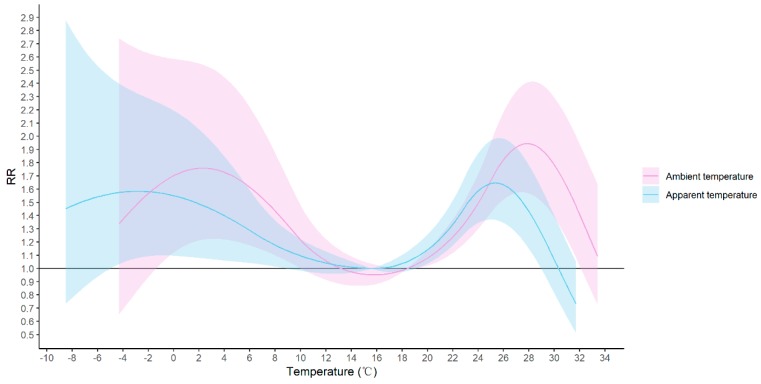
Cumulative effects of ambient temperature and apparent temperature on the incidence of HFMD. The medium value of each variable was selected as the reference.

As a sensitivity analysis, we compared the cumulative effects arising from ambient temperature and apparent temperature within 14 days (Figure 5). We observed a similar ‘M’ shape for both variables. It showed that the apparent temperature had a lower risk with two peaks: one was at −2.8 °C (RR = 1.584, 95% CI: 1.081–2.322) and the other one lay at 25.4 °C (RR = 1.647, 95% CI: 1.367–1.984). We also switched PM_2.5_ to PM_10_ in our analysis and the results remained robust (Figure S2). In addition, we changed the df for long-term trend (6 to 7 df). As a result, the estimated relative risk changed slightly, but did not affect the nonlinear “M” shape previously observed (Figure S3).

## 4. Discussion

In our study, ambient temperature and relative humidity were found to be significantly associated with incidence of HFMD. Lagged effects were observed for extreme temperature and relative humidity. Compared with apparent temperature, ambient temperature seemed to overestimate the potential risk of HFMD. Our study adds to the literature on the short-term association between meteorological factors and the risk of HFMD. Our finding provides more epidemiological evidence for future mechanism studies on how meteorological factors may affect HFMD outbreaks.

This study found that the association between temperature and the incidence of HFMD presented an approximate ‘M’ shape from 1 February 2013 to 1 January 2017, with two peaks at 2.3 °C (RR = 1.760, 95% CI: 1.218–2.542) and 27.9 °C (RR = 1.945, 95% CI: 1.570–2.408). Another study conducted in Wuhan also quantified the influence of temperature on HFMD from 2010 to 2015 using DLNM. The study observed an inverted “V” shape between temperature and HFMD over 14 days and the highest cumulative RR was at 26.4 °C (RR = 2.78, 95% CI: 2.08–3.72) [34]. The effects of temperature during hot days in two studies were similar but there were differences in results regarding low temperature days. The inconsistent results may result from different study periods and model settings, such as lags and df. Both linear and non-linear exposure–response relationships between temperature and HFMD were observed in various cities. Some spotted linear patterns of temperature on HFMD [13,14,16,17], while others noticed the non-linear relationships between temperature and HFMD. For example, a study in Ningbo, China also found an “M” pattern between temperature and HFMD, but the highest risk was at 31 °C [18]. Studies conducted in Chengdu, Guilin, Wuhan, and Guangdong, as well as a Chinese multi-city study, all reported an inverted “V” shape of temperature on the incidence of HFMD [23,26,30,34,35]. In addition to the different model settings, such as lags and covariates, the heterogeneity in the shape of non-linear relationships might result from differences in various climatic characteristics and regional indicators, including geographic location, economy, health care, population, and education [12].

We observed different patterns of the lagged effects due to extreme low and high temperatures. On cold days, two peaks were observed for HFMD at lag3 and lag12. Considering that the incubation period of HFMD is from three to seven days, two different mechanisms regarding the lagged effects were assumed. During lag0 to lag4, a low temperature was statistically significantly associated with increased risk of HFMD. Some studies have found that human immunity could be weakened in cold weather. Short-term exposure to low temperatures may result in a shortened microbial incubation period and lead to the development of infected viruses into diseases [36]. For the peak that occurred at lag12, we think this may be due to changes in people’s behavior caused by exposure to cold weather, which in turn led to greater viral infection potential. For example, on cold days, people wash their hands less frequently, making the most common form of HFMD infection—“contact infection”—possible.

We observed an increased risk of HFMD on hot days as well. The peak of high temperature occurred at lag7, indicating that high temperatures may be associated with more frequent virus attacks. This may be due to the fact that high temperatures facilitate the active replication of the virus, and high temperatures may increase the likelihood of people participating in outdoor activities as well [37]. We observed a downward trend in the risk of HFMD after the peak at 27.9 °C. A previous study showed that strong ultraviolet radiation in hot weather entered inactivated viruses, shortened their circulation, and reduced the likelihood of their transmission back to the host [37,38]. In addition, we found that the lagged effect of low temperature was rapid, and the effects of high temperature was slow, which was consistent with the results of a study in Guilin [23].

In our study, we observed an inverted “V” shape of the association between relative humidity and HFMD incidence. When the relative humidity increased, the risk of HFMD also increased and peaked at a relative humidity of 89.2%. Similar results have been reported in many previous studies [15,20,21,22]. However, studies in Guilin and Huainan showed no significant association between humidity and HFMD [19,23]. Thus far, the mechanism of humidity on HFMD is still unclear, but the findings of previous studies may contribute to the explanation of possible mechanisms. The high-humidity environment is prone to the spread of intestinal viruses, because the virus is more likely to adhere to the surface of objects such as toys, increasing the chance of infection [39,40]. In addition, laboratory experiments have found that the amount of virus in dry air drops faster than in a humid environments [41].

Although many studies have found an increased risk of HFMD at high humidity, the lagged effects we observed in this paper are unique and different from previous findings. In our study, we observed that the highest cumulative effect occurred for a relative humidity of 89.2% at lag12. However, a study in Guangdong showed that the effect of relative humidity peaked at around lag6, and another study showed that the maximum effect of relative humidity occurred at lag11 [42,43]. Climatological, socio-economic, demographic, and infrastructural variations in different areas may explain this discrepancy. As Wuhan is a city with relatively high humidity throughout the year, humans and viruses may not be as sensitive to the change in external relative humidity as they are in other cities.

As humans are exposed to a variety of meteorological factors every day, the ambient temperature may not accurately reflect the human body’s perception of exposure to the external environment. Apparent temperature, a biometeorological indicator that links ambient temperature, humidity, and wind velocity together, is considered to be a more objective index of human response to the thermal environment [31]. Meanwhile, the combined effects of meteorological factors on the transmission of HFMD pathogens may alter the association between ambient temperature and HFMD incidence. Therefore, we applied the apparent temperature rather than the ambient temperature in our sensitivity analysis to verify the robustness of our model. We found a higher risk of HFMD associated with the ambient temperature than the apparent temperature, which was consistent with a study in Spain. The study found that the daily average air temperature had a more severe impact on stroke than the apparent temperature [44]. Our results suggested that humidity and wind speed might modify the effects of temperature and reduce the risk of HFMD. An explanation of this phenomenon would be that during extreme hot days, humans dissipate heat through perspiration. Meanwhile, wind could increase the convective rate, which could accelerate the speed of sweating and mitigate the discomfort of human bodies [45]. In addition, the wind is able to reduce the concentration of virus in the air, thus decreasing the risk of infection [25].

Our research has several strengths. To the best of our knowledge, this is the first study that compared the discrepancy of effects between ambient temperature and apparent temperature on the incidence of HFMD using a distributed, non-linear model. Moreover, considering the potential confounding effects of air pollution on HFMD infection, we adjusted our results by including co-air pollutants PM_2.5_ and SO_2_ in the model to reduce the confounding effects. Third, we proposed two possible pathogeneses of temperature on the incidence of HFMD according to various lagged effects.

Despite the strengths, this ecological study has several limitations. First, we used mean values of meteorological factors and air pollutants from all monitoring stations to replace personal exposure, which may result in misclassification bias. Second, the data on HFMD were collected through passive monitoring, therefore the real amount of HFMD infection may be partly underestimated. Third, as data on personal characteristics such as gender and age are not available, we cannot examine the confounding effects resulting from these factors. Fourth, although we used the apparent temperature to estimate the combined effects of ambient temperature, relative humidity, and wind velocity, we did not look at other meteorological factors, such as sunshine duration and ultraviolet intensity, that may also play a role in the development of HFMD, due to no data being available.

## 5. Conclusions

Our study found significant associations of ambient temperature and relative humidity with HFMD incidence. The non-linear relationship we found between temperature and relative humidity and the incidence of HFMD at different lag days could be applied in the early targeted warning system of infectious diseases, reducing the possible outbreaks and burden of HFMD among sensitive populations. Given the similar transmission and infection mechanisms of airborne viruses, our findings may be applied to early warning systems for other infectious diseases such as influenza and mumps. In addition, we hope that this research can provide a basis for relevant research in the future and the related public health policy-makings.

## Figures and Tables

**Table 1:** Description of meteorological factors, air pollution, and hand, foot and mouth disease (HFMD) cases in Wuhan from 1 February 2013 to 31 January 2017.

Variables	Mean ± SD	Min	25th	50th	75th	Max
Ambient Temperature (°C)	17.29 ± 8.97	−4.32	9.55	18.40	24.80	33.45
Apparent Temperature (°C)	14.62 ± 9.54	−8.55	6.32	15.64	22.77	31.76
Relative Humidity (%)	77.90 ± 10.99	40.66	70.54	78.65	86.09	100.00
Wind Velocity (m/s)	1.73 ± 0.95	0.90	1.04	1.54	2.21	7.16
Atmospheric Pressure (hPa)	1012.93 ± 9.39	994.22	1004.76	1012.59	1020.48	1041.62
Vapor Pressure (hPa)	17.07 ± 8.86	2.25	8.66	16.05	24.66	36.73
PM_10_ (μg/m^3^)	109.98 ± 57.43	12.23	67.90	100.13	140.36	405.97
PM_2.5_ (μg/m^3^)	72.28 ± 48.53	8.24	38.59	60.22	90.86	299.50
SO_2_ (μg/m^3^)	25.31 ± 17.67	3.70	12.90	20.69	32.14	107.58
HFMD (counts/day)	44.80 ± 38.11	0	17	31	64	239

**Table 2:** Spearman correlation between ambient temperature, apparent temperature, relative humidity, wind velocity, atmospheric pressure, vapor pressure, SO_2_, PM_10_, and PM_2.5_.

Variables	Ambient Temperature	Apparent Temperature	Relative Humidity	Wind Velocity	Atmospheric Pressure	Vapor Pressure	PM_10_	PM_2.5_	SO_2_
Ambient Temperature	1								
Apparent Temperature	0.998 *	1							
Relative Humidity	−0.085 *	−0.075 *	1						
Wind Velocity	0.092 *	0.037	−0.124 *	1					
Atmospheric Pressure	−0.908 *	−0.904 *	−0.074 *	−0.135 *	1				
Vapor Pressure	0.963 *	0.965 *	0.166 *	0.061 *	−0.917 *	1			
PM_10_	−0.273 *	−0.263 *	−0.378 *	−0.274 *	0.329 *	−0.376 *	1		
PM_2.5_	−0.496 *	−0.487 *	−0.141 *	−0.233 *	0.488 *	−0.531 *	0.869 *	1	
SO_2_	−0.272 *	−0.268 *	−0.350 *	−0.109 *	0.263 *	−0.361 *	0.533 *	0.551 *	1

* *p* < 0.05.

**Table 3:** The cumulative effects of extreme meteorological factors on the incidence of HFMD at lag0–14.

Percentile	Ambient Temperature		Relative Humidity	
	Value (°C)	RR (95% CI)	Value (%)	RR (95% CI)
1th	−0.1	1.697 (1.113–2.588)	49.2	0.805 (0.597–1.086)
5th	2.9	1.756 (1.224–2.518)	59.0	0.878 (0.746–1.033)
10th	5.0	1.681 (1.202–2.351)	63.5	0.900 (0.769–1.053)
90th	28.7	1.919 (1.530–2.407)	92.2	1.474 (1.261–1.722)
95th	31.0	1.616 (1.221–2.138)	94.7	1.304 (1.113–1.528)
99th	32.5	1.297 (0.916–1.839)	97.5	1.044 (0.841–1.297)

Low temperature and relative humidity: 1st, 5th, and 10th percentiles; High temperature and relative humidity: 90th, 95th, and 99th percentiles. RR: Relative risk.

**Table 4:** The largest cumulative effects of ambient temperature and relative humidity on the incidence of HFMD by lag period.

Lag	Ambient Temperature		Relative Humidity	
	Value (°C)	RR (95% CI)	Value (%)	RR (95% CI)
0–1	−4.3	1.543 (1.047–2.275)	51.3	1.067 (0.966–1.178)
0–2	−4.3	1.893 (1.249–2.869)	60.5	1.073 (0.994–1.157)
0–3	−4.3	2.238 (1.460–3.431)	62.9	1.072 (0.986–1.165)
0–4	−4.3	2.521 (1.623–3.914)	88.1	1.109 (1.020–1.207)
0–5	−4.3	2.691 (1.704–4.249)	88.5	1.165 (1.061–1.278)
0–6	−4.3	2.725 (1.686–4.405)	88.7	1.222 (1.105–1.352)
0–7	−4.3	2.631 (1.576–4.393)	88.8	1.281 (1.149–1.428)
0–8	−4.3	2.444 (1.412–4.230)	88.8	1.339 (1.192–1.504)
0–9	−4.3	2.208 (1.237–3.942)	88.9	1.396 (1.234–1.579)
0–10	−4.3	1.965 (1.076–3.589)	89.0	1.450 (1.273–1.651)
0–11	−1.4	1.795 (1.169–2.757)	89.1	1.498 (1.306–1.719)
0–12	28.3	1.791 (1.467–2.186)	89.2	1.537 (1.329–1.779)
0–13	28.2	1.847 (1.502–2.273)	89.2	1.559 (1.337–1.818)
0–14	27.9	1.945 (1.570–2.408)	89.2	1.553 (1.322–1.824)

RR: Relative risk.

## Supplementary Materials

The following are available online at https://www.mdpi.com/1660-4601/17/2/428/s1, Figure S1: Relative risks of HFMD associated with ambient temperature and relative humidity at lag3, lag7, lag10, and lag14. Figure S2: Cumulative effects of ambient temperature and relative humidity associated with the incidence of HFMD adjusted for PM10 and SO2. Figure S3: Cumulative effects of ambient temperature and relative humidity associated with the incidence of HFMD altering the df for long-term trend.
