# Supplementary material for: Impact of Ambient Temperature and Relative Humidity on the Incidence of Hand-Foot-Mouth Disease in Wuhan, China
Source: Int J Environ Res Public Health. 2020 Jan 8;17(2):428. doi: 10.3390/ijerph17020428 (PMC7013846; doi:10.3390/ijerph17020428)
Supplement: Supplementary file 1 [file ijerph-17-00428-s001.docx]

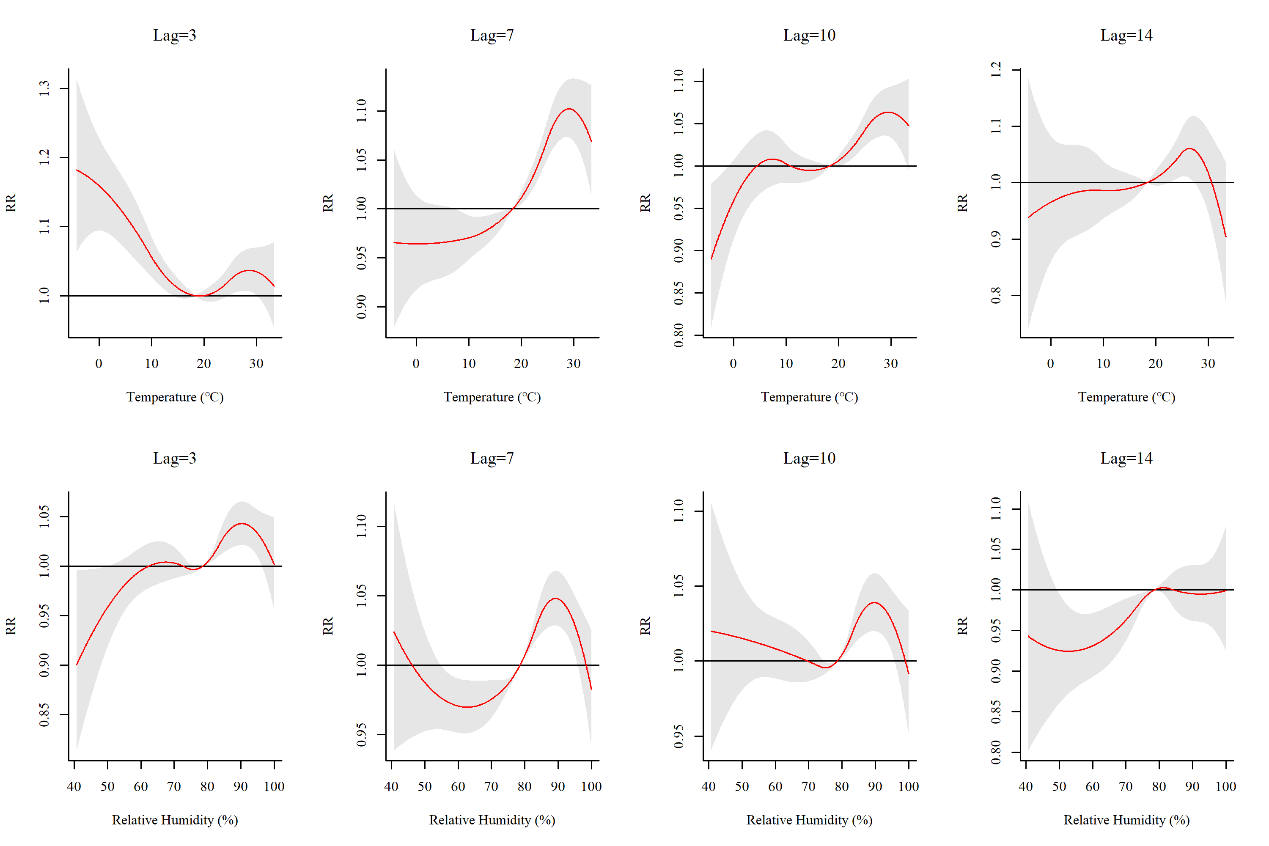


**Figure S1.** Relative risks of HFMD associated with ambient temperature and relative humidity at lag3, lag7, lag10, and lag14.


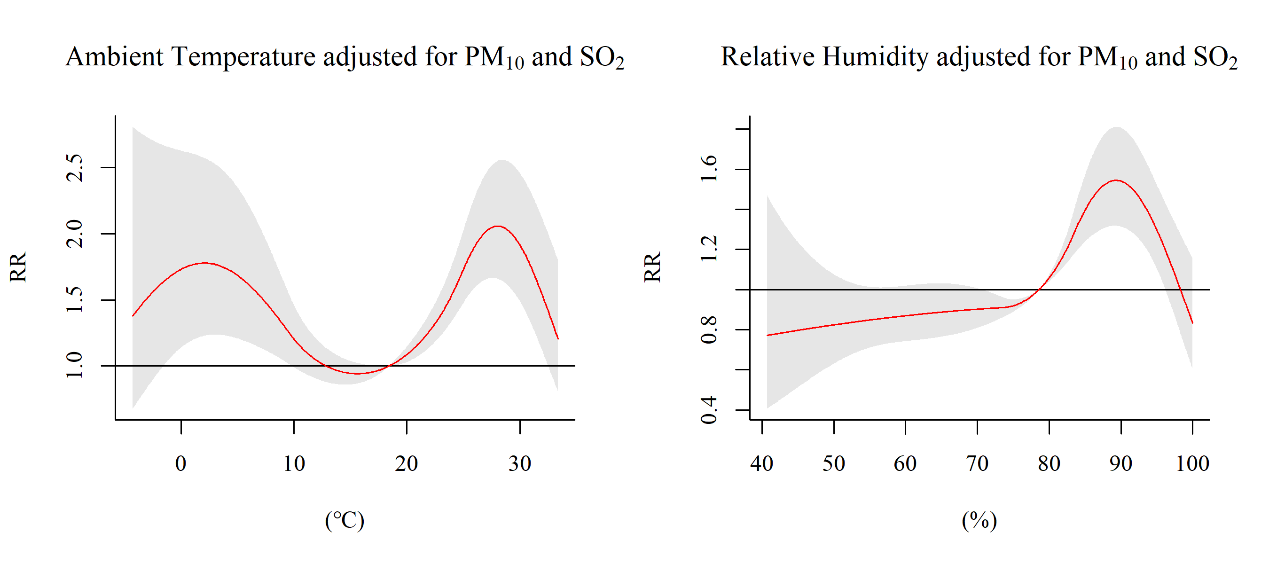


**Figure S2.** Cumulative effects of ambient temperature and relative humidity associated with the incidence of HFMD adjusted for PM_10_ and SO_2_.


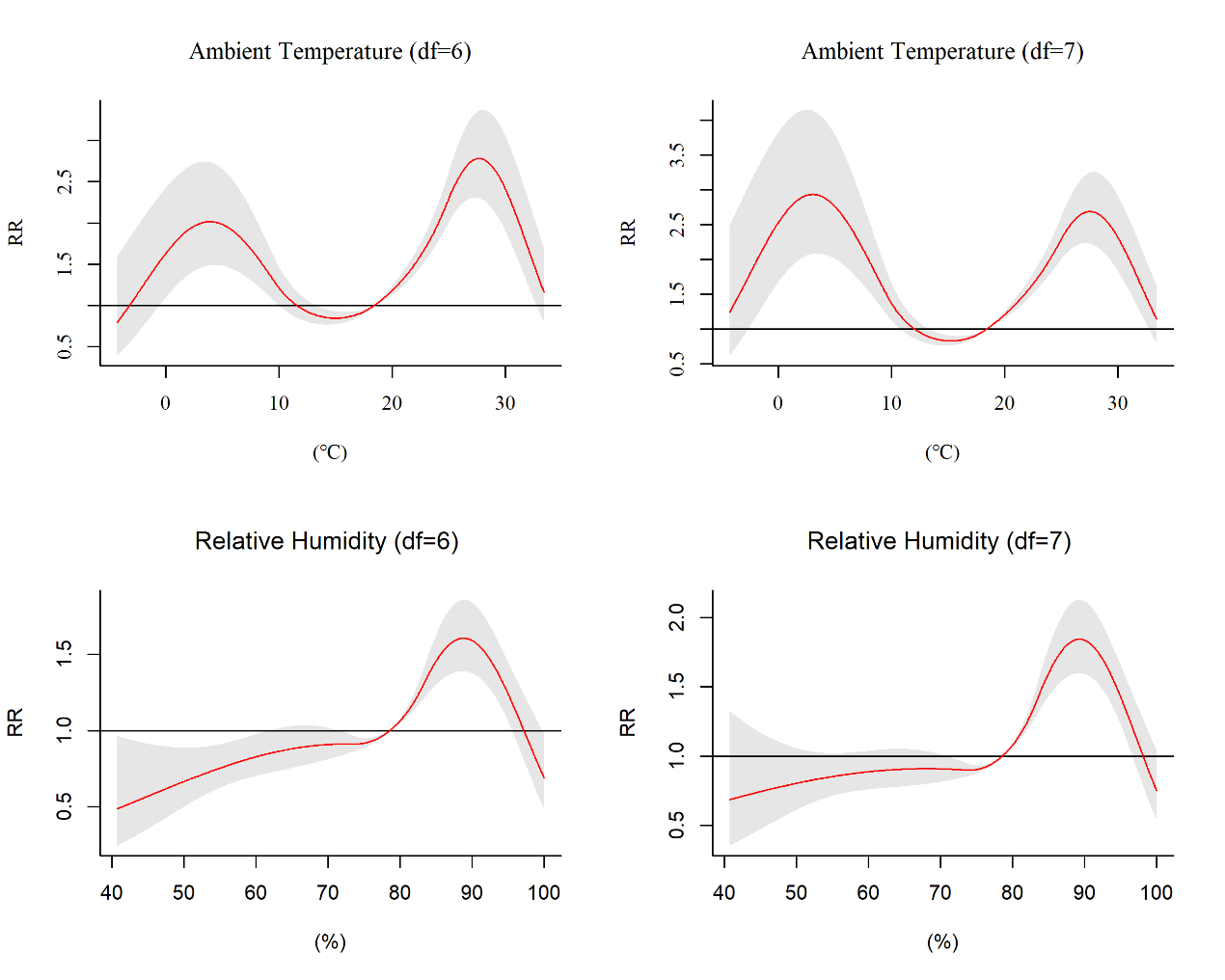


**Figure S3.** Cumulative effects of ambient temperature and relative humidity associated with the incidence of HFMD altering the df for long-term trend.
